# Supplementary material for: Clinical Efficacy of Melon GliSODin® for the Treatment of Aging-Related Dysfunction in Motor Organs—A Double Blind, Randomized Placebo-Controlled Study
Source: J Clin Med. 2022 May 12;11(10):2747. doi: 10.3390/jcm11102747 (PMC9143343; doi:10.3390/jcm11102747)
Supplement: Supplementary file 1 [file jcm-11-02747-s001.zip › jcm-1593772-supplementary.pdf]

CONSORT 2010 checklist of information to include when reporting a randomised trial*

Section/Topic	Item No	Checklist item	Reported on page No
**Title and abstract**			
	1a	Identification as a randomised trial in the title	1
	1b	Structured summary of trial design, methods, results, and conclusions (for specific guidance see CONSORT for abstracts)	1
**Introduction**			
Background and objectives	2a	Scientific background and explanation of rationale	2
	2b	Specific objectives or hypotheses	2
**Methods**			
Trial design	3a	Description of trial design (such as parallel, factorial) including allocation ratio	2
	3b	Important changes to methods after trial commencement (such as eligibility criteria), with reasons	n/a
Participants	4a	Eligibility criteria for participants	2
	4b	Settings and locations where the data were collected	2
Interventions	5	The interventions for each group with sufficient details to allow replication, including how and when they were actually administered	3
Outcomes	6a	Completely defined pre-specified primary and secondary outcome measures, including how and when they were assessed	3-4
	6b	Any changes to trial outcomes after the trial commenced, with reasons	3-4
Sample size	7a	How sample size was determined	4
	7b	When applicable, explanation of any interim analyses and stopping guidelines	n/a
Randomisation:			
Sequence generation	8a	Method used to generate the random allocation sequence	3
	8b	Type of randomisation; details of any restriction (such as blocking and block size)	3
Allocation concealment mechanism	9	Mechanism used to implement the random allocation sequence (such as sequentially numbered containers), describing any steps taken to conceal the sequence until interventions were assigned	3
Implementation	10	Who generated the random allocation sequence, who enrolled participants, and who assigned participants to interventions	3
Blinding	11a	If done, who was blinded after assignment to interventions (for example, participants, care providers, those assessing outcomes) and how	3

	11b	If relevant, description of the similarity of interventions	3
Statistical methods	12a	Statistical methods used to compare groups for primary and secondary outcomes	4
	12b	Methods for additional analyses, such as subgroup analyses and adjusted analyses	4
**Results**			
Participant flow (a diagram is strongly recommended)	13a	For each group, the numbers of participants who were randomly assigned, received intended treatment, and were analysed for the primary outcome	Figure 1
	13b	For each group, losses and exclusions after randomisation, together with reasons	Figure 1
Recruitment	14a	Dates defining the periods of recruitment and follow-up	4-5
	14b	Why the trial ended or was stopped	4
Baseline data	15	A table showing baseline demographic and clinical characteristics for each group	5
Numbers analysed	16	For each group, number of participants (denominator) included in each analysis and whether the analysis was by original assigned groups	Figure 1
Outcomes and estimation	17a	For each primary and secondary outcome, results for each group, and the estimated effect size and its precision (such as 95% confidence interval)	6
	17b	For binary outcomes, presentation of both absolute and relative effect sizes is recommended	6
Ancillary analyses	18	Results of any other analyses performed, including subgroup analyses and adjusted analyses, distinguishing pre-specified from exploratory	6
Harms	19	All important harms or unintended effects in each group (for specific guidance see CONSORT for harms)	6
**Discussion**			
Limitations	20	Trial limitations, addressing sources of potential bias, imprecision, and, if relevant, multiplicity of analyses	7
Generalisability	21	Generalisability (external validity, applicability) of the trial findings	7
Interpretation	22	Interpretation consistent with results, balancing benefits and harms, and considering other relevant evidence	6-7
**Other information**			
Registration	23	Registration number and name of trial registry	2
Protocol	24	Where the full trial protocol can be accessed, if available	UMIN portal
Funding	25	Sources of funding and other support (such as supply of drugs), role of funders	8

*We strongly recommend reading this statement in conjunction with the CONSORT 2010 Explanation and Elaboration for important clarifications on all the items. If relevant, we also recommend reading CONSORT extensions for cluster randomised trials, non-inferiority and equivalence trials, non-pharmacological treatments, herbal interventions, and pragmatic trials. Additional extensions are forthcoming: for those and for up to date references relevant to this checklist, see www.consort-statement.org.

Supplementary Tables

Supplementary Table S1. A comparison of baseline values concerning oxidative markers, antioxidants, and inflammatory markers; blood biochemistry; bone metabolism; body composition; and motor function between the placebo (n = 22) and Melon GliSODin® (n = 24) groups

	Placebo	Melon GliSODin®	P-value
**Oxidative markers, antioxidants, and inflammatory markers**			
**Plasma pentosidine, µg/mL**	0.06 (0.01)	0.06 (0.03)	0.09#
**MDA, µmol/L**	0.78 (0.47)	0.74 (0.33)	0.82#
**dROMs, U. CARR**	353.14 (66.62)	325.52 (74.11)	0.39#
**BAP, µmol/L**	2316.05 (376.68)	2275.00 (429.38)	0.74
**Serum SOD activity, U/mL**	5.08 (3.85)	3.56 (2.35)	0.11#
**Serum GPx activity, U/mL**	0.29 (0.09)	0.33 (0.10)	0.14
**hsCRP, ng/mL**	605.09 (524.95)	380.75 (245.32)	0.30#
**TNFα, pg/mL**	1.18 (0.91)	1.36 (1.37)	0.88#
**IL-6, pg/mL**	1.14 (0.59)	1.31 (0.76)	0.50#
**Blood biochemistry**			
**TP, g/dL**	7.00 (0.41)	7.00 (0.40)	1.00
**Alb, g/dL**	4.25 (0.25)	4.33 (0.19)	0.29#
**AST, U/L**	21.68 (5.17)	24.54 (11.45)	0.39#
**ALT, U/L**	15.45 (4.63)	18.17 (11.66)	0.71#
**BUN, mg/dL**	15.41 (4.54)	16.79 (2.38)	0.08#
**Cre, mg/dL**	0.64 (0.09)	0.62 (0.15)	0.23#
**Bone metabolism**			
**ucOC, ng/mL**	4.40 (2.18)	4.53 (2.50)	0.98#
**Osteocalcin, ng/mL**	18.44 (5.30)	19.59 (7.11)	0.54
**Total P1NP, ng/mL**	52.11 (24.96)	51.25 (18.11)	0.89
**TRACP-5b, mU/dL**	307.00 (121.27)	298.54 (87.41)	0.79
**Ca, mEq/L**	9.46 (0.39)	9.46 (0.43)	0.97

**Pi, mEq/L**	3.63 (0.37)	3.75 (0.46)	0.33
**Body composition**			
**YAM (lumbar), %**	93.59 (11.33)	89.46 (15.69)	0.33
**YAM (hip joint), %**	85.14 (14.05)	85.75 (13.59)	0.66
**Whole YAM, %**	96.59 (7.95)	94.71 (8.71)	0.50
**SMI, kg/m^2**	6.15 (0.69)	5.95 (0.50)	0.32
**Fat mass, g**	18327.77 (4835.02)	19180.71 (5275.74)	0.76#
**Non-fat mass, g**	33408.5 (3495.84)	33073.25 (3845.89)	0.69
**Body fat percentage, %**	35.05 (6.91)	36.85 (6.17)	0.45
**Motor function**			
**Maximum torque: Left, Nm**	71.80 (15.27)	60.35 (26.31)	0.08
**Maximum torque: Right, Nm**	72.48 (16.75)	62.98 (24.55)	0.14
**Grip: Left, kg**	22.90 (3.21)	21.86 (3.82)	0.21#
**Grip: Right, kg**	23.38 (3.89)	22.50 (4.31)	0.30#
**TUG, s**	7.49 (0.99)	7.63 (2.21)	0.52#
**6 minute walk, m**	544.68 (95.83)	569.25 (139.24)	0.48#
**Two-step test, distance/height**	208.05 (22.60)	205.79 (25.74)	0.59#
**Stand-up test, score**	4.45 (1.14)	3.83 (0.76)	0.049#

Values are shown as mean (SD); # *P*-values obtained using Mann-Whitney U tests; all other using Student's *t*-tests; $^*P < 0.05$;
Abbreviations: Alb, albumin; ALT, alanine aminotransferase; AST, aspartate aminotransferase; BAP, biological antioxidant potential; BUN, blood urea nitrogen; Ca, calcium; Cre, creatinine; dROMs, diacron reactive oxygen metabolites; GPx, glutathione peroxidase; hsCRP, high-sensitivity C-reactive protein; IL-6, interleukin-6; MDA, malondialdehyde; P1NP, type I procollagen-N-propeptide; Pi, phosphorus; SMI, Skeletal Muscle Mass Index; SOD, superoxide dismutase; TNFα, tumor necrosis factor-α; TP, total protein; TRACP-5b, tartrate-resistant acid phosphatase 5b; TUG, timed up-and-go test; ucOC, undercarboxylated osteocalcin; YAM, Young Adult Mean

Supplementary Table S2. A comparison of values concerning circulating concentrations of oxidative markers, antioxidants, and inflammatory markers; blood biochemistry; bone metabolism; body composition; and motor function between the placebo (n = 22) and Melon GliSODin® (n = 24) groups after 6 months

Outcomes	6-month outcome			Baseline to 6 month change		
	Placebo	Melon GliSODin®	P value	Placebo	Melon GliSODin®	P-value
**Oxidative markers, antioxidants, and inflammatory markers**						
**Plasma pentosidine, µg/mL**	0.06 (0.01)	0.06 (0.04)	0.50#	0.00 (0.01)	0.00 (0.03)	0.72
**MDA, µmol/L**	0.83 (0.48)	0.70 (0.36)	0.76#	0.05 (0.31)	-0.04 (0.21)	0.27
**dROMs, U.CARR**	362.41 (86.16)	319.29 (77.91)	0.08	9.27 (59.08)	-8.71 (52.72)	0.28
**BAP, µmol/L**	2330.55 (289.30)	2278.63 (430.03)	0.64	14.5 (320.03)	11.91 (399.98)	0.98
**Serum SOD activity, U/mL**	5.63 (5.28)	3.53 (2.08)	0.36#	0.54 (3.30)	-0.03 (0.47)	0.40
**Serum GPx activity, U/mL**	0.31 (0.09)	0.33 (0.11)	0.39	0.02 (0.07)	0.00 (0.06)	0.39
**hsCRP, ng/mL**	1207.00 (2867.31)	3213.08 (14163.43)	0.13#	601.90 (2589.60)	2832.33 (14021.15)	0.47
**TNFα, pg/mL**	1.03 (1.07)	0.79 (0.49)	0.27#	-0.15 (1.43)	-0.58 (1.21)	0.28
**IL-6, pg/mL**	1.25 (0.92)	1.65 (2.20)	0.34#	0.10 (0.80)	0.34 (2.05)	0.61
**Blood biochemistry**						
**TP, g/dL**	7.00 (0.44)	6.96 (0.38)	0.79	0.0 (0.37)	-0.04 (0.31)	0.75
**Alb, g/dL**	4.26 (0.22)	4.32 (0.25)	0.41	0.00 (0.29)	-0.01(0.23)	0.83
**AST, U/L**	22.68 (4.43)	22.08 (4.80)	0.68#	1.00 (3.89)	-2.46 (13.46)	0.25
**ALT, U/L**	16.68 (4.04)	15.58 (6.42)	0.50	1.22 (4.84)	-2.58 (12.91)	0.20
**BUN, mg/dL**	15.23 (3.68)	15.75 (3.00)	0.60	-0.18 (2.02)	-1.04 (3.03)	0.27
**Cre, mg/dL**	0.64 (0.09)	0.63 (0.09)	0.62	-0.00 (0.04)	0.01 (0.11)	0.72
**Bone metabolism**						
**ucOC, ng/mL**	4.65 (2.25)	5.38 (3.08)	0.71#	0.25 (1.20)	0.85 (1.25)	0.10
**Osteocalcin, ng/mL**	18.46 (5.40)	20.51 (6.70)	0.26	0.03 (2.65)	0.92 (2.43)	0.24
**Total P1NP, ng/mL**	51.84 (19.22)	56.45 (18.36)	0.41	-0.27 (11.47)	5.2 (10.41)	0.10
**TRACP-5b, mU/dL**	329.41 (100.05)	310.79 (99.94)	0.53	22.4 (81.43)	12.3 (46.56)	0.60
**Ca, mEq/L**	9.52 (0.32)	9.58 (0.33)	0.56	0.05 (0.39)	0.12 (0.37)	0.58
**Pi, mEq/L**	3.74 (0.41)	3.83 (0.29)	0.36	0.10 (0.33)	0.08 (0.44)	0.83
**Body composition**						

**YAM (Lumbar), %**	93.00 (12.15)	89.50 (16.56)	0.42	-0.59 (2.52)	0.04 (3.26)	0.47
**YAM (hip joint), %**	85.09 (12.68)	85.29 (13.90)	0.96	-0.05 (3.37)	-0.46 (2.23)	0.62
**Whole YAM, %**	96.55 (7.61)	95.17 (9.36)	0.59	-0.05 (1.50)	0.46 (2.19)	0.37
**SMI, kg/m^2**	6.10 (0.62)	5.97 (0.54)	0.45	-0.04 (0.26)	0.03 (0.28)	0.40
**Fat mass, g**	18270.77 (4927.36)	18752.50 (5100.40)	0.86$^{\#}$	-57.00 (1159.49)	-428.21 (1044.13)	0.26
**Non-fat mass, g**	33068.50 (3577.72)	33390.17 (4001.66)	0.78	-340.00 (872.62)	316.92 (1143.64)	0.04*
**Body fat percentage, %**	35.23 (7.15)	36.12 (5.67)	0.64	0.17 (1.50)	-0.72 (1.64)	0.06
**Motor function**						
**Maximum torque: Left, Nm**	74.24 (16.22)	65.52 (19.55)	0.17	2.44 (8.16)	5.2 (16.60)	0.49
**Maximum torque: Right, Nm**	74.19 (16.14)	66.75 (19.80)	0.17	1.71 (7.22)	3.76 (12.26)	0.50
**Grip: Left, kg**	23.15 (3.55)	21.72 (4.22)	0.16$^{\#}$	0.25 (2.30)	-0.14 (2.82)	0.61
**Grip: Right, kg**	24.27 (3.31)	22.00 (5.13)	0.09	0.89 (2.90)	-0.50 (2.92)	0.11
**TUG, s**	7.13 (0.84)	7.28 (1.32)	0.87$^{\#}$	-0.35 (1.08)	-0.35 (1.88)	0.99
**6-minute walk, m**	575.05 (80.64)	575.46 (84.78)	0.92$^{\#}$	30.4 (87.36)	6.21 (118.25)	0.44
**Two-step test, distance/height**	213.45 (23.72)	201.29 (28.33)	0.23	0.04 (0.15)	-0.03 (0.14)	0.14
**Stand-up test, score**	4.23 (0.92)	3.63 (0.97)	0.038*	-0.23 (0.97)	-0.21 (0.51)	0.93

Values are shown as mean (SD); $^{\#}$ *P*-values obtained using Mann-Whitney U tests; all other using Student's *t*-tests; $^{*}P < 0.05$;
Abbreviations: Alb, albumin; ALT, alanine aminotransferase; AST, aspartate aminotransferase; BAP, biological antioxidant potential; BUN, blood urea nitrogen; Ca, calcium; Cre, creatinine; dROMs, diacron reactive oxygen metabolites; GPx, glutathione peroxidase; hsCRP, high-sensitivity C-reactive protein; IL-6, interleukin-6; MDA, malondialdehyde; P1NP, type I procollagen-N-propeptide; Pi, phosphorus; SMI, Skeletal Muscle Mass Index; SOD, superoxide dismutase; TNFα, tumor necrosis factor-α; TP, total protein; TRACP-5b, tartrate-resistant acid phosphatase 5b; TUG, timed up-and-go test; ucOC, undercarboxylated osteocalcin; YAM, Young Adult Mean

Supplementary Data

Details of the participants who did not complete the study

Number of participants who started the study

Fifty patients were to be included and gave their informed consent. Of these, 46 (melon glisodin group: n=24, placebo group: n=22) completed the study and four were excluded during the study period.

Details of the participants who were excluded

a) A 75-year-old woman in the Melon GliSODin® group (locomo grade 1)

The participant underwent blood collection, exercise, and other tests on an initial occasion and then returned home. However, after re-reading the instructions for participation in the clinical study, she became more anxious and chose not to participate further. There was no particular problem with the initial blood sampling and motor function assessments, and she did not take either of the treatments.

Height: 142 cm, Body weight: 53 kg, BMI: 26.3 kg/m2

Locomo 25, score: 4, JKOM, score: 43, Chalder fatigue scale, score: 6, VRS, score: 2, RDQ, score: 2

Stand-up test, score: 3, Two-step test, distance/height: 1.17

b) A 54-year-old woman in the placebo group

When the participant first underwent assessment of her motor function, she lost her balance, fell, and bruised her head. Head computed tomography imaging showed a lacunar infarction but no traumatic changes. Therefore, she was excluded from the study before commencing the treatment. She used to run a marathon on a daily basis and the lesion was suspected to be associated with that exercise.

No data: Height, Body weight, BMI, Locomo 25, JKOM, Chalder fatigue scale, VRS, RDQ, Stand-up test, Two-step test

c) A 74-year-old woman in the placebo group (locomo grade 2)

During her participation in the study, her hearing deteriorated, and on examination a tumor was found on an auditory nerve. There were no adverse events that could be related to the placebo.

Height: 150 cm, Body weight: 48.1 kg, BMI: 21.4 kg/m2

Locomo 25, score: 17, JKOM, score: 38, Chalder fatigue scale, score: 14, VRS, score: 2, RDQ, score: 5

Stand-up test, score: 4, Two-step test, distance/height: 1.47

e) A 54-year-old woman in the placebo group (locomo grade 2)

The participant experienced no change in her knee pain during the first 2 months of the study; therefore, she elected not to continue the study. No adverse events were recorded that could be related to the placebo use.

Height: 162 cm, Body weight: 63 kg, BMI: 24.0 kg/m2

Locomo 25, score: 21, JKOM, score: 54, Chalder fatigue scale, score: 15, VRS, score: 3, RDQ, score: 4

Stand-up test, score: 3, Two-step test, distance/height: 1.23
